# EuroSCORE Models in a Cohort of Patients with Valvular Heart Disease and a High Prevalence of Rheumatic Fever Submitted to Surgical Procedures

**DOI:** 10.1371/journal.pone.0118357

**Published:** 2015-02-25

**Authors:** Ricardo Casalino, Flávio Tarasoutchi, Guilherme Spina, Marcelo Katz, Antonio Bacelar, Roney Sampaio, Otavio T. Ranzani, Pablo M. Pomerantzeff, Max Grinberg

**Affiliations:** 1 Heart Institute—University of São Paulo Medical School, São Paulo, Brazil; 2 Hospital Israelita Albert Einstein, São Paulo, Brazil; CUNY, UNITED STATES

## Abstract

**Objectives:**

Epidemiological differences can be found between Brazilian and European valvular heart disease patients. The prevalence of heart valve diseases due to rheumatic disease is significantly higher in the Brazilian compared with the European population. Therefore, they could have different risks during and after cardiac surgery. The aim of this study was to evaluate the applicability of the additive and logistic EuroSCORE and EuroSCORE II in a cohort of high-risk patients with valvular heart disease of predominantly rheumatic aetiology submitted to surgery.

**Methods:**

Between 1 February and 30 December 2009, 540 consecutive patients scheduled for valvular heart surgery were included in this study. In this set of patients, we examined the performance of the additive, logistic, and EuroSCORE II models for predicting in-hospital mortality. Calibration of each model was assessed by comparing predicted and observed in-hospital mortality and by the goodness of fit of the Hosmer-Lemeshow chi-square test. Discrimination performance of the model was evaluated with the receiver operating characteristic (ROC) curve analysis.

**Results:**

The mean age was 56 ± 16 years, 50.6% were female, and the mortality rate was 16.0% (6.0% in elective surgery and 34.0% in emergency/urgency surgery). Mortality rates were estimated according to the additive and logistic EuroSCORE and EuroSCORE II at 6.1%, 8.7%, and 4.3%, respectively. The AUC was 0.76 (95% confidence interval [95% CI] 0.70–0.81) for the additive EuroSCORE, 0.76 (95% CI 0.70–0.81) for the logistic EuroSCORE and 0.81 (95% CI 0.76–0.86) for EuroSCORE II. Hosmer-Lemeshow goodness-of-fit statistics were P = 0.52, P = 0.07, and P = 0.12 for additive, logistic EuroSCORE, and EuroSCORE II.

**Conclusions:**

In this cohort of Brazilian patients with valvular heart disease submitted to surgical procedure, the EuroSCORE models had a good discriminatory capacity; however, the calibration was compromised because of an underestimation of the mortality rate.

## Introduction

Preoperative risk stratification is essential to making sound surgical decisions. Risk scoring systems have been developed to predict mortality after cardiac surgery in adults. The European System for Cardiac Operative Risk Evaluation (EuroSCORE), developed in European states is designed to predict the 30-day mortality rate of patients undergoing cardiac surgery [1–2]. This risk scoring system has been validated with good results in Australian, European, North American, and Japanese cohorts. EuroSCORE has also been applied to predict additional useful endpoints, including long-term mortality, length of stay in intensive-care units, and the costs of cardiac surgery [3–12]. A recent update (EuroSCORE II) is currently being validated with results published worldwide [13]. Such risk-scoring systems are more applicable when the preoperative patients’ characteristics and treatment profiles are comparable with those on which the system was developed. Although used in many Brazilian and in other developing countries institutions, there have been very few local validations of EuroSCORE [14], especially when considering patients who undergo valvular heart surgery.

Brazilian patients have a higher prevalence of rheumatic disease than European patients do, in addition to being relatively younger, having more than one compromised valve, and exhibiting a lower prevalence of coronary heart disease and other age-related comorbidities [15]. However even in industrialized countries, cases of rheumatic heart disease are frequent with similar epidemiological findings [16].

In an era of transcatheter aortic heart valve replacement when indications of valve replacement are based heavily on risk scores, the importance of score validation increases. Risk scores also support the comparison of outcomes between institutions and surgeons, facilitating the communication necessary for clinical research.

At our center, we had used the original EuroSCORE model as an important reference for the risk prediction in heart valve surgery. However, the predictive value of EuroSCORE models remained unknown and there is no previously published report on the applicability of the EuroSCORE models to heart valve surgery patients in Brazil or in other developing countries with similar profile of patients.

Therefore, the aim of this study was to evaluate the performance of additive, logistic, and EuroSCORE II in predicting in-hospital mortality in a cohort of patients with valvular heart disease and a predominant rheumatic aetiology submitted to surgical procedure in a tertiary hospital that receives patients from all over Brazil.

## Materials and Methods

### Patients

**Fig 1:**
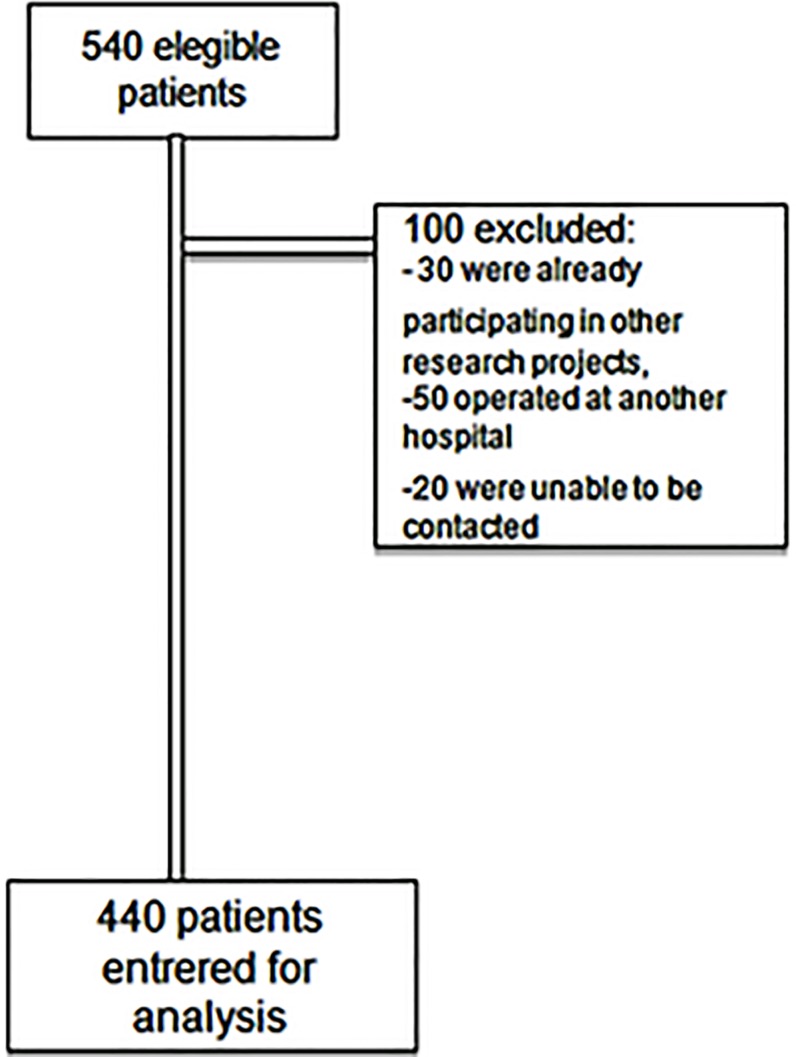
Flowchart: patients’ enrolment.

Between 1 February and 30 December 2009, 540 consecutive patients with valvular heart disease (mitral, aortic, tricuspid, and pulmonary) underwent surgery at the Heart Institute of the University of São Paulo. Inclusion criteria were age above 18 years and the following procedures: isolated valve surgery: mitral, aortic, tricuspid, and pulmonary surgery with prosthetic replacements or repairs and combined valvular surgery (more than one valve), and coronary surgery combined with valvular heart disease. Exclusion criteria were participation in other research projects, not undergoing surgery at our hospital despite being scheduled for it, and other such things. 100 patients were excluded as follows: 30 were participating in other research projects, 50 underwent surgery at another hospital, and 20 were lost to follow-up after hospital discharge. Ultimately, 440 patients were included in the study (Fig. 1). The protocol was approved by the institutional Review Board of the University of São Paulo School of Medicine (Protocol number 0155/10) and written informed consent was obtained from all the subjects.

### Study protocol

The following data were collected before and after surgery: patient demographics, patient risk factors, surgery details (which surgery and surgical events), length of hospital stay, in-hospital and 30-day mortality. The additive and logistic EuroSCORE algorithms were calculated according to the published guidelines (http://www.euroscore.org) for the entire patient sample prior to the time of surgery. The calculation of each patient’s score was conducted by the same two physicians. In October 2011, after the publication and availability of the EuroSCORE II online calculator, the EuroSCORE II algorithm was retrospectively calculated for all 440 patients. All three EuroSCORE models were used to analyze all patients, and the subgroups of rheumatic and non-rheumatic patients as well as elective and emergency surgery were analyzed separately. There were no missing data for EuroSCORE calculation.

### Statistical analysis

Continuous variables are expressed as mean ± standard deviation (SD) or median with an interquartile range. Categorical variables are expressed as absolute and relative frequencies. The differences between patient groups were analysed using two-sample t tests for continuous variables or the chi-squared test for categorical variables. The area under the curve (AUC) was used to evaluate the predictive performance (model discrimination) of the EuroSCORE models, and the AUCs were compared using the Hanley and McNeil method [17]. The calibration (statistical precision) was assessed using the Hosmer-Lemeshow goodness-of-fit statistic [18], and the chi-square statistic measured the differences between expected and observed outcomes over tertiles of risk. The entire cohort was divided into tertiles according to score values from our sample. Therefore, the cohort was clustered into three groups: the lowest score (low-risk group), intermediate score (medium-risk group) and high score (high-risk group). Data analysis was performed using STATA/SE 11.0 (StataCorp LP, College Station, TX, USA). A P value of less than 0.05 was considered statistically significant.

## Results

Patient characteristics are reported in Table 1. The mean age was 56±12 years, and 50.6% of the patients were female. The most common aetiology of valvular heart disease was rheumatic disease (56.1%), followed by calcific degeneration (15.4%) and mitral valve prolapse (12.0%). Less prevalent aetiologies are listed in Table 1.

**Table 1:** Baseline characteristics of the entire group, separated into rheumatic and non-rheumatic patients.

	Total: N = 440	Rheumatic N = 247	Non-rheumatic N = 193
Age—yrs (mean±SD)	56 ± 16	51 ± 12	62 ± 11
Female	223 (50.6%)	150 (60.3%)	74 (38.3%)
Chronic comorbidities			
Arterial hypertension	205 (46.5%)	102 (41.3%)	30 (15.0%)
Diabetes mellitus	61 (13.8%)	25(10.2%)	35 (18.3%)
Atrial fibrillation	187 (42.5%)	131(53.1%)	54(27.0%)
Aetiology			
Rheumatic valve disease	247 (56.1%)		
Calcification	70 (15.9%)		
Prolapse	53 (12.0%)		
Infective endocarditis	42 (9.5%)		
Ischemic complication	14 (3.1%)		
Bicuspid valve	9 (2.0%)		
Aortic aneurism	8 (1.8%)		
Valvar disease			
Aortic valve stenosis	124 (28.1%)	50 (20.2%)	77 (40.1%)
Mitral valve regurgitation	113 (25.6%)	42 (17.0%)	70 (36.2%)
Mitral prosthesis dysfunction	53 (12.0%)	47 (19.1%)	12 (6.3%)
Mitral valve stenosis	48 (10.9%)	52 (21.3%)	2 (1.1%)
Aortic valve regurgitation	38 (8.6%)	23 (9.4%)	14 (7.4%)
Aortic prosthesis dysfunction	31 (7.0%)	18 (7.3%)	12 (6.2%)
Combined mitral lesion	10 (2.2%)	8 (3.3%)	3 (1.3%)
Combined aortic lesion	6 (1.3%)	4 (1.7%)	1 (0.5%)
Tricuspid regurgitation	4 (0.9%)	3 (1.4%)	1 (0.5%)
Others	11 (2.5%)	8 (1.7%)	4 (2.2%)
CABG combined surgery	45 (10.2%)	22 (8.9%)	23 (12.1%)
Preoperative echography			
Impaired left ventricular function	103 (23.4%)	55 (22.3%)	50 (25.9%)
Segmental dysfunction	44 (10.0%)	15 (6.3%)	45 (13.5%)
Pulmonary hypertension	224 (50.9%)	142 (57.5%)	80 (41.3%)
Impaired right ventricular function	98 (22.2%)	59 (23.7%)	21(10.9%)
Diastolic dysfunction	119 (27.0%)	34 (13.8%)	61 (31.7%)
NYHA classification			
I	13 (2.9%)	4 (1.7%)	10 (5.2%)
II	97 (22.0%)	40 (16.3%)	54 (28.0)%
III	266 (6.4%)	170 (68.8%)	98 (50.8%)
IV	62 (14.0%)	58 (13.5%)	31 (16.0%)

SD—standard deviation; CABG—cardiac artery bypass graft, NYHA—New York Heart Association.

There was a predominance of mitral valve disease (51.8%), as expected in a population with a high prevalence of rheumatic heart disease, and a high incidence of reoperations (31.0%), most often related to bioprosthesis dysfunction. A low percentage of patients underwent combined valvular and coronary artery bypass graft (CABG) surgery (10.2%). The patient’s comorbidities are also listed in Table 1, with atrial fibrillation documented in 42.5% of patients, diabetes in 13.8% patients, and hypertension in 46.5% of patients.

### EuroSCORE analysis

The main differences between the present cohort and the original EuroSCORE cohort were age, sex, previous cardiac surgery, pulmonary hypertension, and active endocarditis (Table 2) [19]. The frequency of comorbidities, such as chronic pulmonary disease, extracardiac arteriopathy, and neurological disease was low at 2.0%. Thirty-one percent of the study population had undergone previous valvular heart surgery. The majority had undergone only one previous surgery (25.0%), whereas a smaller proportion had undergone more than one surgery (6.0%). There were differences between the EuroSCORE II population and our own population sample. The EuroSCORE II patients were older (64.6 ± 12.5), less female (30.9%), more likely to have chronic pulmonary disease (10.7%). The prevalence of endocarditis was low (2.2%) in EuroSCORE II population.

**Table 2:** EuroSCORE variables in the Brazilian cohort and the original EuroSCORE database.

ES Variables	Brazilian ES	Original ES	P value
Age (years)	56 ± 16	62.5 ± 10.7	<0.001
Female (%)	50.6	27.8	<0.001
Chronic pulmonary disease (%)	2.0	3.9	0.04
Extracardiac arteriopathy (%)	1.4	11.3	<0.001
Neurological disease (%)	2.2	1.4	0.25
Previous cardiac surgery (%)	31.0	7.3	<0.001
Creatinine > 200mmol/L (%)	3.2	1.8	0.07
Active endocarditis (%)	9.5	1.1	<0.001
Critical preoperative state (%)	5.8	4.1	0.05
Unstable angina (%)	2.7	8	<0.001
Moderate LV dysfunction (%)	18.9	25.6	0.002
Severe LV dysfunction (%)	4.5	5.8	0.47
Recent myocardial infarction (%)	4.0	9.7	<0.001
Pulmonary hypertension (%)	21.2	2	<0.001
Emergency (%)	4.1	4.9	0.43
Postinfarct septal rupture (%)	-	0.2	>0.99*
Surgery on thoracic aorta (%)	0.9	2.4	0.04
Other surgery than CABG (%)	100	36.4	<0.001

SD—standard deviation

CABG—coronary artery bypass grafting

ES—EuroSCORE, LV—left ventricular

Table adapted from: Yap CH1, Reid C, Yii M, Rowland MA, Mohajeri M, Skillington PD, Seevanayagam S, Smith JA. Validation of the EuroSCORE model in Australia. Eur J Cardiothorac Surg. 2006 Apr;29(4):441–6.

A chi-squared test was used to compare EuroSCORE variables in the Brazilian cohort and the original EuroSCORE database.

Pulmonary hypertension was observed in 21.0% of the study population using the additive and logistic EuroSCORE definition (>60 mm Hg), whereas using the EuroSCORE II definition, 30.2% of the study population had moderate pulmonary hypertension (31–55 mm Hg), and 20.7% had severe pulmonary hypertension (>55 mm Hg).

A total of 4.0% of the patients in the study underwent emergency surgery, but using EuroSCORE II definitions, 32.5% of the patients in the study were scheduled for surgery after reaching the emergency room (urgent surgery). The main reason for emergency surgery was acute heart failure secondary to valvular disease followed by prosthesis dysfunction and endocarditis. Before undergoing heart valve surgery, these patients required clinical treatment for an average of 7±3 days.

The means of the additive and logistic EuroSCOREs were 6±3 and 8.6±10.3, respectively. The mean value of EuroSCORE II was 4.2±5.9. A total of 132 patients (30.0%) were in the low-risk group (logistic EuroSCORE mean: 2.18±0.59, EuroSCORE II mean: 0.8±0.2), 168 patients (38.1%) were in the medium-risk group (logistic EuroSCORE mean: 5.2±0.1, EuroSCORE II mean: 2.2±0.7) and 140 patients (31.8%) were in the high-risk group (logistic EuroSCORE mean: 18.5±13.1, EuroSCORE II mean: 9.4±7.9) (Table 3).

**Table 3:** Risk tertiles, calibration, and chi-squared tests to compare predicted and observed mortality of EuroSCORE models.

Model	Risk tertiles	Mean	SD	Predicted	Observed	H-L (P)	χ^2^ test(P)
					N (%)		
ES II	First (0–4) 331 patients	0.8	0.2	1	6 (4.1)	0.40	0.12
	Second (5–7) 60 patients	6.0	2.0	3	18 (12.2)	0.10	0.001
	Third (8+) 49 patients	9.4	7.9	14	47 (32)	0.88	<0.001
	Total	4.2	5.9	19	71 (16.1)	0.12	<0.001
Additive ES	First (0–4) 132 patients	2.9	0.7	4	4 (2.9)	0.41	>0.99
	Second (5–7) 168 patients	5.7	0,7	10	22 (13.3)	0.01	0.02
	Third (8+) 140 patients	9.8	2.1	14	45 (32.1)	0.16	<0.001
	Total	6.2	3.0	27	71 (16.1)	0.52	<0.001
Logistic ES	First (0–4) 204 patients	2.1	0.5	3	7 (4.8)	0.95	0.19
	Second (5–7) 79 patients	5.2	1.1	8	17 (11.6)	0.65	0.06
	Third (8+) 157 patients	18.5	13.1	27	47 (32)	0.27	0.007
	Total	8.6	10.4	38	71 (16.1)	0.07	0.001

H-L—Hosmer-Lemeshow

SD—standard deviation

ES—EuroSCORE

χ^2—^chi-squared

EuroSCORE means of each risk tertile and calibration by the Hosmer-Lemeshow test. The chi-squared statistic measured the differences between expected and observed outcomes over tertiles of risk of all three models.

**Fig 2:**
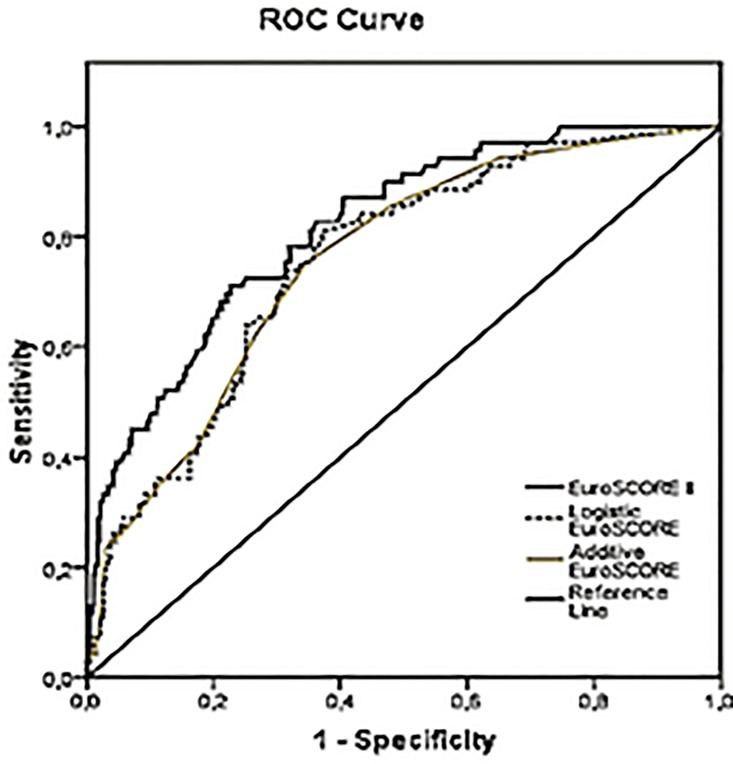
Receiver-operating characteristic (ROC) curve of EuroSCORE models.

The AUCs of the logistic and additive EuroSCOREs and the EuroSCORE II were 0.76 [95% confidence interval (CI) 0.70–0.81], 0.76 (95% CI 0.70–0.81), and 0.81 (95% CI 0.76–0.86), respectively, for the entire cohort (Fig. 2). There was no difference when comparing the AUCs using the Hanley and McNeil method (P = 0.9).

When separated by aetiology, the discriminatory ability included AUCs of 0.76 (95% CI 0.69–0.84), 0.77 (95% CI 0.70–0.85) and 0.79 (95% CI 0.71–0.86) in the rheumatic aetiology and 0.78 (95% CI 0.70–0.85), 0.77 (95% CI 0.69–0.85) and 0.84 (95% CI 0.77–0.90) in the nonrheumatic aetiology for additive and logistic EuroSCOREs and EuroSCORE II, respectively (Table 4).

**Table 4:** Subgroup analysis—Discriminatory ability of EuroSCORE models when split into rheumatic and non-rheumatic aetiologies.

Model	AUC	95%CI	P value	H-L (P)
Rheumatic etiology				
ES additive	0.76	0.69–0.84	<0.001	0.42
ES logistic	0.77	0.70–0.85	<0.001	0.01
ES II	0.79	0.71–0.86	<0.001	0.10
Non-rheumatic aetiology				
ES additive	0.782	0.70–0.85	<0.001	0.25
ES logistic	0.773	0.69–0.85	<0.001	0.07
ES II	0.840	0.77–0.90	<0.001	0.26

AUC—area under the receiver operating characteristic curve

H-L—Hosmer-Lemeshow

ES—EuroSCORE

When separated by surgery urgency, the discriminatory ability included AUCs of 0.70 (95% CI 0.61–0.78), 0.70 (95% CI 0.61–0.78) and 0.76 (95% CI 0.68–0.84) in the emergency surgery and 0.79 (95% CI 0.71–0.88), 0.79 (95% CI 0.71–0.87) and 0.80 (95% CI 0.74–0.86) in the elective surgery for additive and logistic EuroSCOREs and EuroSCORE II, respectively (Table 5).

**Table 5:** Subgroup analysis—Discriminatory ability of EuroSCORE models when split into emergency and elective surgery.

Model	AUC	95%CI	P value	H-L (P)
Emergency surgery				
ES additive	0.70	0.61–0.78	<0.001	0.18
ES logistic	0.70	0.61–0.78	<0.001	0.20
ES II	0.76	0.68–0.84	<0.001	0.40
Elective surgery				
ES additive	0.79	0.71–0.88	<0.001	0.16
ES logistic	0.79	0.71–0.87	<0.001	0.16
ES II	0.80	0.74–0.86	<0.001	0.17

AUC—area under the receiver operating characteristic curve

H-L—Hosmer-Lemeshow

ES—EuroSCORE

Model calibration with Hosmer-Lemeshow goodness-of-fit statistics reveal P = 0.52, P = 0.07, and P = 0.12 for additive and logistic EuroSCOREs and EuroSCORE II, respectively (Table 3). Analysis of the tertiles of risk revealed a tendency to underestimate mortality in the medium and high-risk groups for all three scores, with differences among the three scores (Table 3). When dividing our population, the cut-off value for high risk (EuroSCORE >7) was higher than the original EuroSCORE cohort (EuroSCORE >5). So using original EuroSCORE cut-offs, the majority of the patients were in the in the high-risk category.

The predicted mortality for the entire group according to the additive and logistic EuroSCOREs and EuroSCORE II was 6.2%, 8.5%, and 4.3%, respectively. The overall mortality was 16.0%, 6.0% (21 patients) in elective surgery and 34.0% (39 patients) in emergency/urgency surgery, all of which were in-hospital mortalities.

## Discussion

This study examined a heterogeneous group of patients with valvular heart disease submitted to cardiac surgery; the group was significantly different from the original EuroSCORE cohort, which had predominantly coronary disease. However, our sample was close to the EuroSCORE II population, whom had significantly valve procedure (46.0%), with aortic predominance and degenerative aetiology.

Some reasons could explain our higher mortality rates. As was expected in the Brazilian valvular cohort and predominantly rheumatic population submitted to surgery, there was a much higher incidence of younger females with pulmonary hypertension due to the prevalence of mitral valve disease. Mitral valve disease, particularly mitral stenosis, frequently leads to severe pulmonary hypertension [20], a risk factor for poor outcomes. Indeed, Barbieri et al. showed that pulmonary hypertension is a frequent complication of significant mitral regurgitation due to flail leaflet and is associated with major outcome implications that approximately double the risk of death and heart failure after diagnosis [21].

Female gender is other independent predictor of mortality after cardiac surgery according to the EuroSCORE models. Doenst et al. conducted a retrospective analysis of 1567 patients who had undergone combined valve and coronary surgery and concluded that although women have a higher preoperative risk profile than men have when undergoing combined valve and CABG surgery, their long-term survival rates are similar [22].

The peculiarities of valvular patients were also expressed in a higher incidence of infective endocarditis (9.5%), a complication typically observed in this cohort of patients. Revilla et al. demonstrated that endocarditis is a condition associated with high mortality, particularly for those who need urgent surgery, which explains the high mortality rate of our cohort [23].

Atrial fibrillation has also been identified as a risk factor for mortality and morbidity associated with valve surgery, as it may cause low cardiac output during the postoperative period or predispose the patient to thromboembolic events [24]. In our series, atrial fibrillation was observed in 42.5% of the patients, although it was not correlated with mortality, as de Almeida Brandão et al. reported [25].

Given that this cohort was younger, markedly less atherosclerotic disease was observed, as expressed in the lower incidence of recent myocardial infarction (4.0% vs. 9.7%; P<0.001), unstable angina (2.7% vs. 8%; P<0.001), and extracardiac arteriopathy (1.4% vs. 11.3%; P<0.001), all of which were 10 times more frequent in the original EuroSCORE cohort.

The large number of reoperations (31.0%) caused by the predominantly rheumatic aetiology can be explained by the young age of most of the patients at the first operation. Likewise, if valve replacement was needed, most of the patients had a bioprosthesis implanted. The institutional preference for bioprosthesis use is linked to the poor social conditions of the patients. Most live far from medical facilities and thus are not able to engage in the ambulatory monitoring of oral anticoagulation. The high prevalence of bioprosthesis implantation contributes to the high incidence of reoperations due to bioprosthesis failure over time. Piehler et al. identified the number of previous valve operations as an independent predictor of hospital mortality, and de Almeida Brandão et al. reported an overall hospital mortality rate of 10.9%, with a rate of 3.8% for reoperations involving the mitral position and 11.1% for those involving the aortic position [25–26]. Although the mortality rate appeared to be higher for first reoperations than for second or third reoperations, this difference was not significant and may be due to other variables that influence mortality such as ventricular function and renal insufficiency.

Two variables related to the surgical procedure and one variable related to the patient raise concern about the applicability of EuroSCORE in the study population. The first was emergency surgery (after referral, carried out before the beginning of the next day). At the Heart Institute, 32.5% of patients who had surgery after arriving decompensated in the emergency room. These patients displayed no greater risk according to EuroSCORE, because they did not meet the criteria for emergency surgery. The EuroSCORE II population had 18.5% urgent and 4.3% emergent surgery, very similar with our population. The main reason for emergency surgery in our population was a high degree of heart failure (NYHA III/IV) secondary to valvular disease, prosthesis dysfunction, and endocarditis.

The second variable was the type of surgery. In the initial EuroSCORE database, surgery types were subdivided into isolated CABG or other than CABG that was independent of the number of procedures. With the publication of EuroSCORE II, these confounding variables were corrected [13]. Surgery types were subdivided into elective, urgent, emergency, and salvage. Urgent and emergency patients are those who have not been electively admitted for operation but required surgery based on the current admission for medical reasons. Salvage patients are those requiring cardiopulmonary resuscitation en route to the operating theatre or before the induction of anesthesia. These cases do not include cardiopulmonary resuscitation following the induction of anesthesia. Procedure types were further separated into categories such as CABG, valve repair or replacement, replacement of part of the aorta, repair of a structural defect, maze procedures, and the resection of a cardiac tumor.

The third patient-related variable was pulmonary hypertension. In the original EuroSCORE cohort, the categorization of this variable using the cut-off of 60 mm Hg masked the variable’s importance as a risk predictor, because many, and thus, despite showing elevated pulmonary pressure, are not categorized by EuroSCORE as being at higher risk. However, with the publication of EuroSCORE II, pulmonary pressure was categorised as either moderate (systolic pressure 31–55 mm Hg) or severe (>55 mm Hg). These variable modifications presented in EuroSCORE II were intended to avoid the risk of underestimation and possibly explain the best discriminative capacity of this new model.

Despite the epidemiological differences and characteristics of our high-risk population, the EuroSCORE models provided good discriminatory ability (AUC>0.75). The dataset used to develop the EuroSCORE II model included patients from four centers in Brazil and a number of other centres outside of Europe. As expected, EuroSCORE II showed the greatest accuracy in all patients and also when the cohort was split into rheumatic/non-rheumatic etiologies, however it was not statistically different from both EuroSCORE models. When comparing the predicted and observed mortality rates using the chi-squared test, the mortality was underestimated by all three EuroSCORE models in medium- and high-risk tertiles (P<0.05). In the low-risk tertile, no statistically significant difference was observed between the predicted and observed mortalities (P>0.05).

Risk scoring systems should not be the only tools used to preoperatively predict risk. The clinical experience and the individual characteristics of each patient should be an essential part of surgical decision making (the heart-team concept).

Epidemiological and comorbidity differences represented by the high prevalence of pulmonary hypertension, infective endocarditis, atrial fibrillation, previous cardiac surgery, and high rates of surgical urgency were decisive in our results and probably influence the calibration. These variables had greater weight in predicting risk in our population compared with the EuroSCORE population. All three scores have good discriminatory capacity; however, the observed mortality rates were higher than the predicted mortality. This poor calibration limits the use of these scores in clinical practice. For best applicability recalibration could be a solution; however, this strategy is out of the scope of this study. Another strategy is the use of locally developed risk score, such as the recently reported InsCor. [27]

## Limitations

This study included a heterogeneous group of patients with valvular heart disease who were scheduled for surgery in a single center. After initial enrolment, 50 of the patients underwent surgery at other hospitals for medical reasons and thus were excluded from this analysis. The EuroSCORE II values were calculated retrospectively using database information. Finally, the sample size for the subgroup analysis was not adequately powered to definitive conclusions, since the initial calculation was based on the analysis of entire cohort.

In conclusion, in this cohort of valvular heart disease patients submitted to cardiac surgery, the EuroSCORE models had a good discriminatory capacity. However, calibration was compromised by an underestimation of mortality rates. Observed mortality was higher than predicted mortality in all subgroups with all the three scores.
